# Quantum LFSR Structure for Random Number Generation Using QCA Multilayered Shift Register for Cryptographic Purposes

**DOI:** 10.3390/s22093541

**Published:** 2022-05-06

**Authors:** Hyun-Il Kim, Jun-Cheol Jeon

**Affiliations:** 1 Department of Robotics Engineering, Daegu Gyeongbuk Institute of Science & Technology, Dalseong-gun, Daegu 42988, Korea; hyunil89@dgist.ac.kr; 2 Department of Convergence Science, Kongju National University, Gongju 32588, Korea

**Keywords:** cryptography, random number generator, linear feedback shift register, quantum-dot cellular automata, cell interaction

## Abstract

A random number generator (RNG), a cryptographic technology that plays an important role in security and sensor networks, can be designed using a linear feedback shift register (LFSR). This cryptographic transformation is currently done through CMOS. It has been developed by reducing the size of the gate and increasing the degree of integration, but it has reached the limit of integration due to the quantum tunneling phenomenon. Quantum-dot cellular automata (QCA), one of the quantum circuit design technologies to replace this, has superior performance compared to CMOS in most performance areas, such as space, speed, and power. Most of the LFSRs in QCA are designed as shift registers (SR), and most of the SR circuits proposed based on the existing QCA have a planar structure, so the cell area is large and the signal is unstable when a plane intersection is implemented. Therefore, in this paper, we propose a multilayered 2-to-1 QCA multiplexer and a D-latch, and we make blocks based on D-latch and connect these blocks to make SR. In addition, the LFSR structure is designed by adding an XOR operation to it, and we additionally propose an LFSR capable of dual-edge triggering. The proposed structures were completed with a very meticulous design technique to minimize area and latency using cell interaction, and they achieve high performance compared to many existing circuits. For the proposed structures, the cost and energy dissipation are calculated through simulation using QCADesigner and QCADesigner-E, and their efficiency is verified.

## 1. Introduction

Current CMOS technology is a basic technology used in the digital and analog electronics industry. CMOS continues to be developed by reducing the size of the gate and increasing integration, but it is approaching the limit of integration due to the quantum tunneling phenomenon [1]. To solve this problem, nano-circuit design technologies such as quantum-dot cellular automata (QCA) are being developed to replace CMOS. QCA circuit technology is a next-generation digital nano circuit design technology with many advantages such as low power consumption and fast switching speed [2]. In QCA, circuits made based on digital logic can be redesigned and used based on QCA, and many digital circuits in CMOS such as logical and arithmetic operators have been proposed using QCA [3,4,5,6,7,8,9,10].

A multiplexer (Mux) is a combination circuit that is essential in most digital circuit designs, and it determines an output line according to an input selection value. Mux can be used effectively to create storage space such as D-latch or D flip-flop (F/F), and can be implemented easily. Circuits can be implemented using basic logical operators such as AND, OR, and NOT, and various circuits designed using fault tolerance and cell interaction are emerging. Efforts to minimize latency and area have continued by designing not only coplanar structures, but also multilayered structures [11,12,13,14,15,16,17,18,19].

Using D-latch or D-F/F, the shift register (SR) can be designed and developed into the linear feedback shift register (LFSR) structure we want to make. A latch simply stores and outputs a value, whereas F/F has a structure in which the output value is determined by a change in the clock. There is also a negative-edge-triggered structure that generates an output when the clock changes to 0 and a positive-edge-triggered structure that produces an output when the clock changes to 1, and there is a dual-edge-triggered structure that enables both. In addition, D-F/F with a reset function has been proposed to increase efficiency, but it has resulted in the degradation of delay time and space efficiency [20,21,22,23,24,25,26]. 

As SRs are widely used in digital circuits such as memory circuits, computer output and input ports, and counter configurations, many studies have been conducted based on QCA [27,28,29,30,31,32,33]. SRs have been generally proposed by connecting blocks made based on D-latch or D-F/F. However, there is a problem of delay of the clock signal transmitted to each block. Therefore, improved circuits were suggested so that the clock signal can be transmitted equally to each block. Additionally, a multilayered structure was used to solve the spatial complexity problem. Conventional SR circuits have various problems such as structural parts, signal noise, time and space complexity, and clock synchronization. In this paper, we propose a 2-to-1 Mux and a D-F/F with a multilayer structure with cell interaction. The proposed structures solve various existing problems and constitute an efficient *n*-bit SR. 

In addition, we designed the LFSR structure, which plays an important role in the random number generator [34,35,36]. Various LFSR structures have been proposed in the past, but a large amount of wasted area was used for wiring, or due to an effort to reduce such space, various problems of latency and signal transmission or energy dissipation increased [36,37,38,39]. The proposed structures not only solve the area and latency problems mentioned above, but also minimize energy dissipation, which is very important in designing a large quantum circuit. The contributions of this work can be itemized as follows.

- A multilayered 2-to-1 Mux using cell interaction is proposed. Additionally, an optimized D-latch is proposed using the Mux.
- By connecting the proposed D-latch, a 4-bit SR with modularity and scalability is proposed using a multilayered structure.
- A three-input XOR gate is connected to the proposed SR to complete the 4-bit LFSR structure, and a dual-edge trigged LFSR structure is additionally proposed.
- The proposed structures and the structures of existing papers were compared, the accuracy of design and operation was checked and compared using QCADesigner [40], the latency and required area were checked, and the cost was calculated.
- Finally, the proposed LFSR structure was compared with the best existing structures by additionally calculating energy dissipation using QCADesigner-E [41].

In this paper, we propose an LFSR structure for random number generation. This paper is structured as follows. Section 2 describes the basic knowledge of QCA and previously proposed 2-to-1 Muxes, D-latches, SRs, and LFSRs. Section 3 presents a multilayered 2-to-1 Mux and extends it to implement D-latch. In addition, the SR structure is made by connecting D-latch to several blocks, the three-input XOR gate is connected, and the LFSR structure is proposed. Additionally, an LFSR structure capable of dual-edge triggering is also proposed. Section 4 compares and analyzes the performance of the proposed circuits and the existing circuits in area, latency, and energy dissipation. Finally, we conclude in Section 5.

## 2. Related Works

### 2.1. Background of QCA

**Figure 1:**
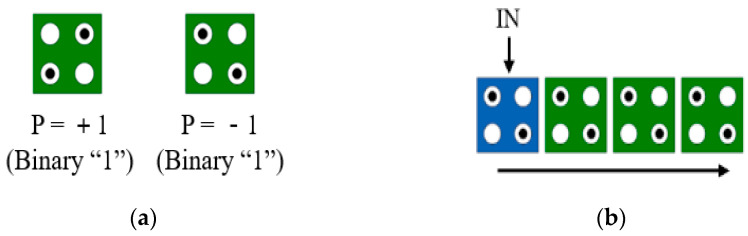
QCA basic concept: (**a**) two possible polarizations; (**b**) wiring.

A quantum cell, a basic component of QCA, has four quantum dots and two electrons. Each electron is positioned diagonally to each other due to Coulomb repulsion [2]. These electrons have two types of arrangements according to the input signal, and each type has a polarization of +1 (binary logic 1) or −1 (binary logic 0) as shown in Figure 1a. Additionally, QCA cells can be placed adjacent to each other and used as QCA wiring. If the polarization state of one cell is specified, the adjacent cells change to the same polarization to lower the energy between cells, as shown in Figure 1b, and the signal will be transmitted [3,4,5,6,7]. 

**Figure 2:**
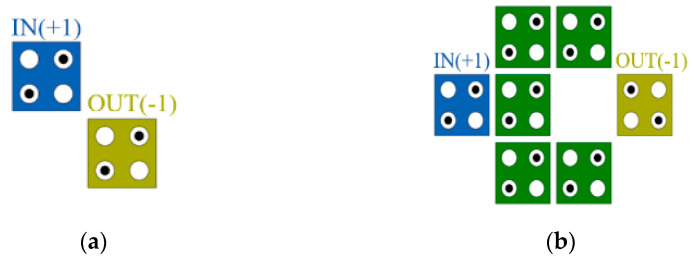
Inverters: (**a**) week inverter; (**b**) robust inverter.

Figure 2 shows a representative inverter gate among the basic gates composed of QCA cells. In the inverter gate, when a value is input from the input cell IN, the output cell OUT changes to the opposite polarization and outputs it. The inverter in QCA can be implemented in the form of the weak inverter in Figure 2a, which transmits a weak signal although the required area is small, and the robust inverter in Figure 2b, which takes up a relatively large area although the signal is transmitted strongly [8,9,10].

### 2.2. Multilayer Structure

**Figure 3:**
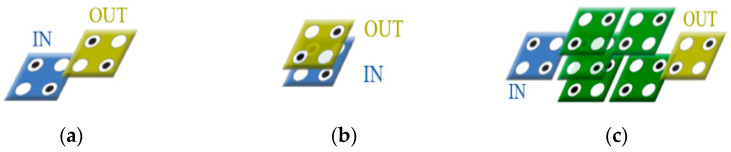
Multi-layer structures: (**a**) diagonal line; (**b**) vertical line; and (**c**) multi-layer robust inverter.

The QCA circuit is classified into a planar structure using only one layer and a multilayer structure using multiple layers. Unlike the planar structure, the multilayer structure has an interlayer interaction. As shown in Figure 3a, when cells are connected diagonally, the signal is transmitted as it is in the existing planar structure, but as shown in Figure 3b the cells are connected vertically. It has the property of an inverter in which the signal of the cell is inverted [11]. Cells connected vertically have higher signal strength than cells connected with diagonal lines, and efficient circuit design can be achieved using this property.

The multilayer structure is closer to the electrons than the planar structure, so the signal strength is strong and it only requires a small area. As shown in Table 1, when the conventional planar structure inverter is changed to a multilayered inverter as shown in Figure 3, the weak inverter of Figure 3b has higher signal strength and a much smaller area than the robust inverter of Figure 2b. Using these characteristics to design a multilayer structure can have several advantages over designing a planar structure. However, due to the complexity of the design and the difficulty of implementation, very delicate work is required.

### 2.3. Previous QCA Multiplexers and D-Latch

A Mux is a circuit that selects one of several input signals and delivers the selected input to an output line. This is used in many circuits such as D-latch, registers, and RAM cells, and a D-latch can be designed by creating a loop section using a Mux. Table 2 is the truth table of the 2-to-1 Mux, which has three input signals, S, A, and B, and one output, OUT. The input value S is a selection signal, and depending on whether it is 0 or 1, the input value A or B is outputted, respectively. 

**Figure 4:**
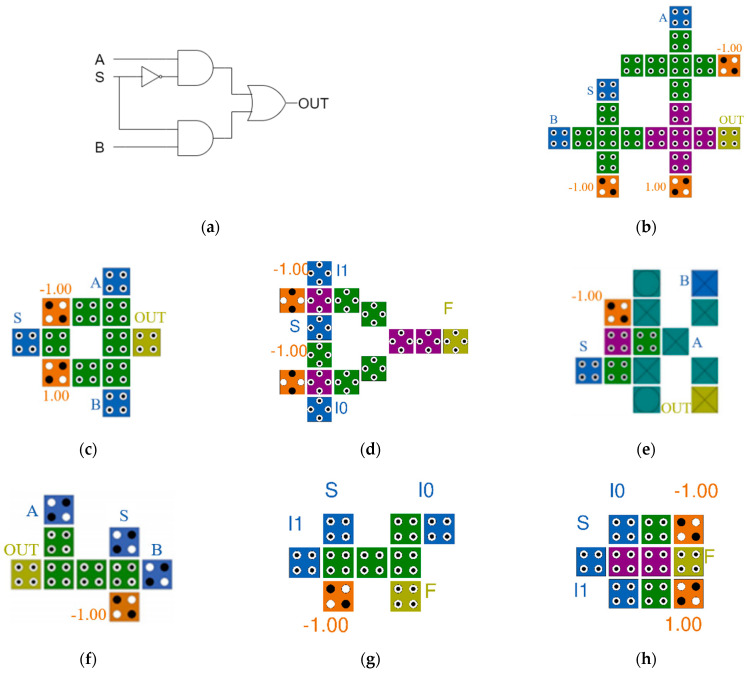
Previous 2-to-1 Muxes: (**a**) a logic diagram of 2-to-1 multiplexer; (**b**) B. Sen et al.’s [12]; (**c**) M. N. Asfestani et al.’s [13]; (**d**) D. Ajitha et al.’s [14]; (**e**) M. Mosleh’s [15]; (**f**) A. H. Majeed et al.’s [16]; (**g**) A. H. Majeed’s [17]; and (**h**) S. S. Ahmadpour’s [18].

Figure 4a is a logic diagram of a 2-to-1 Mux. A circuit is built using one NOT gate, two AND gates, and one OR gate. In Figure 4, S denotes selection lines, A (or I0) and B (or I1) denote input lines, OUT (or F) denotes output lines, and orange cells denote fixed cells with −1 or +1. Figure 4b through Figure 4h show previously proposed QCA 2-to-1 Mux circuits [12,13,14,15,16,17,18]. In Figure 4b, a weak inverter is used for NOT operation and three majority gates are used for AND and OR operations according to the format of the basic logic diagram in Figure 4a. Figure 4d,e was designed using a majority gate, and each was designed using a rotated cell and a multilayer structure, respectively. The remaining circuits were designed to minimize complexity by using cell interaction instead of implementing logical operations.

The principle of Figure 4c is that when the value of S is +1, the signal of fixed cell (−1) is weakened and the signal of fixed cell (+1) is strengthened, so that the signal of B, which is close to fixed cell (+1), is output. The first two circuits designed for cell interaction are a good way to reduce the number and area of cells, but due to the limitation of the planar structure there is a disadvantage in that the area increases when designing a large circuit using this Mux as an element. Figure 4e is a previously proposed multilayer Mux circuit. Although it can be made smaller than Figure 4b, it is operated on the first and third layers and used as a simple connection line that transmits only signals on the second layer so that it does not have spatial superiority compared to Figure 4f through Figure 4h.

Table 3 is the truth table of D-latch, which is a circuit that can store and maintain 1-bit information and is a basic element of a sequential circuit. The D-latch has an input signal D and a clock input CLK, and one output. D-Latch gives the result value of the Mux as an input value again. When CLK is 1, the result value is changed, and when CLK is 0, the previous value is output.

**Figure 5:**
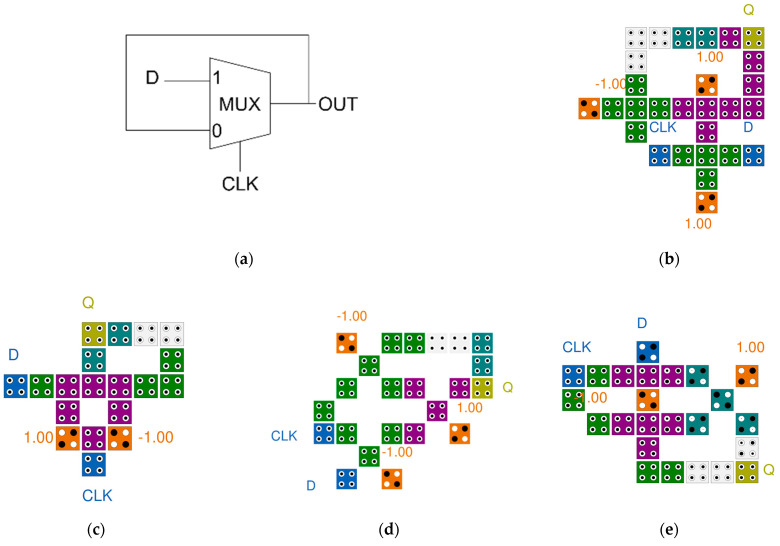

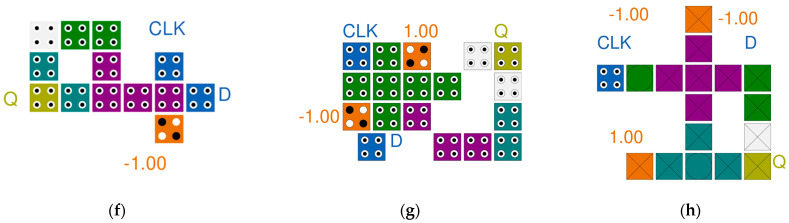
Previous D-latches: (**a**) a logic diagram of D-latch; (**b**) M. M. Abutaleb’s [20]; (**c**) M. G. Roshan et al.’s [21]; (**d**) T. N, Sasamal et al.’s [22]; (**e**) J. C. Jeon’s [23]; (**f**) A. H. Majeed et al.’s [24]; (**g**) Z. Song et al.’s [25]; and (**h**) D. K. Seo et al.’s [26].

Figure 5a shows a logic diagram of D-latch. Figure 5b,e show that wiring is added to the Mux circuit based on the majority vote, while Figure 5c,f,g confirm that the wiring is connected to the Mux by cell interaction. Figure 5d uses a rotated majority gate, and Figure 5h shows the completed D-latch using a multilayered structure. As shown in Figure 5, D-latch is completed by adding wiring to the existing Mux circuit. Therefore, it can be seen that the performance of the D-latch circuit is determined by how efficiently and well the Mux circuit is designed.

### 2.4. Previous QCA Shift Register and LFSR Structure

**Figure 6:**
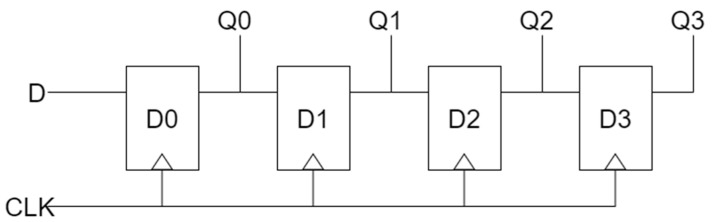
A logic diagram of 4-bit shift register.

An SR is a circuit that temporarily stores binary information and transfers information left or right. The SR consists of a number of D-latches. The output of each latch is connected to the input of the next latch. According to the first clock input, one bit of binary information is input to the SR, and the previously stored information is moved along with the next clock input. Q0 to Q3 can check the output value of each D-latch of every clock. A logic diagram of the *n*-bit shift register is shown in Figure 6.

**Figure 7:**
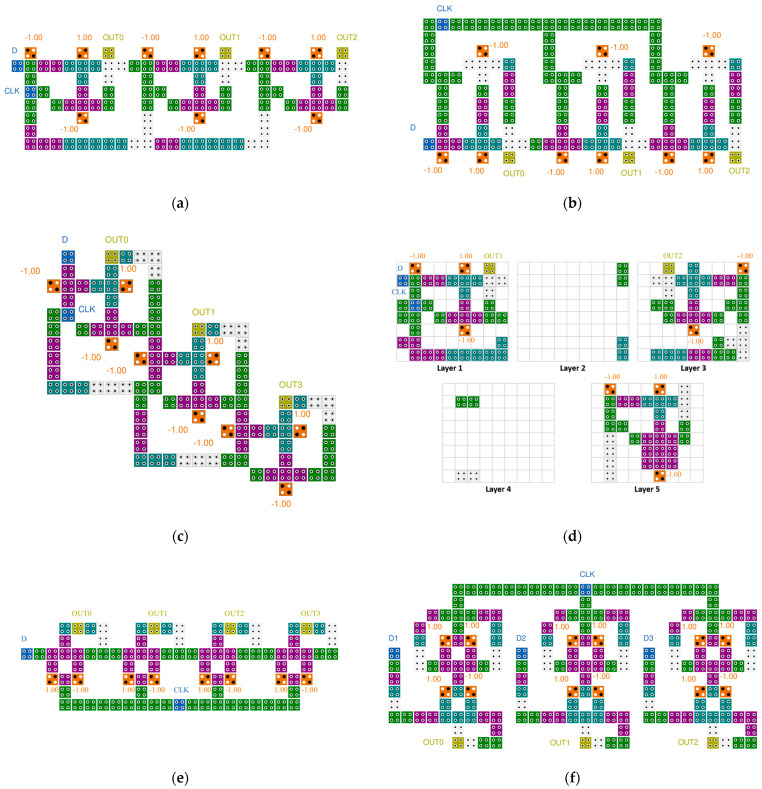
Typical 3~4 bit SRs: (**a**) J. C. Das’s [28]; (**b**) M. N. Divshali et al.’s [29]; (**c**) M. Abdullah-Al-Shafi et al.’s [30]; (**d**) T. Li et al.’s [31]; (**e**) M. G. Roshan et al.’s [32]; and (**f**) S. Fan et al.’s [33].

Figure 7 shows the previously proposed QCA 3- or 4-bit SR circuits. In papers [28,30], a 3-bit serial SR was proposed by arranging three D-latch blocks, but there was a problem that the clock was sequentially delivered to each block, resulting in a long delay. To solve this problem in papers [29,32,33], the clocks were synchronized. In paper [31], an effort was made to reduce the area of the SR by using a multilayer structure, but there was a problem in that the implementation complexity was surpassed by using five layers and the development cost increased. In addition, as each layer has a different structure, it lacks modularity and scalability. In papers [28,29,30,31], the circuits had a SISO structure with serial input and output, but the circuit in [32] developed a SIPO structure that allowed simultaneous output, and the circuit in [33] developed a PIPO structure that allowed simultaneous output as well as simultaneous input.

**Figure 8:**
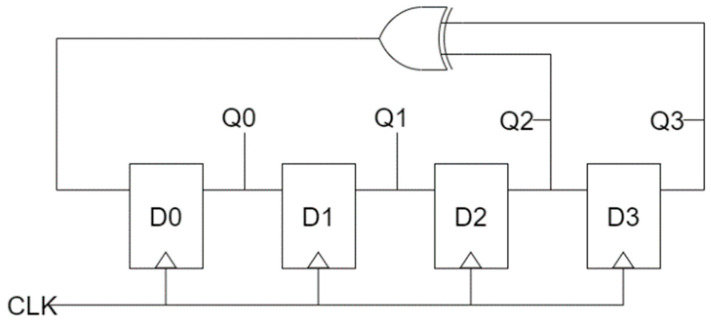
A logic diagram of 4-bit LFSR.

LFSR can be used for a random number generator (RNG), a cryptographic technology that plays an important role in security, and is a binary stream generator applied to various stream ciphers [34,35,36]. The LFSR is designed to have high periodicity and good statistical properties, and Figure 8 shows a 4-bit logic diagram of the LFSR structure. The initial values of the D-latch are shifted to the right every clock, and Q2 and Q3 are input to D0 by performing an XOR operation every clock. For example, if the initial value is 1010, the following values are calculated in the order of 1101, 1110, 1111, 0111, 0011, 0001, 1000, 0100, 0010, 1001, 1100, 0110, 1011, and 0101. With the exception of 0000, it has a maximum periodicity to obtain 15 combinations of all 4-bit random numbers.

**Figure 9:**
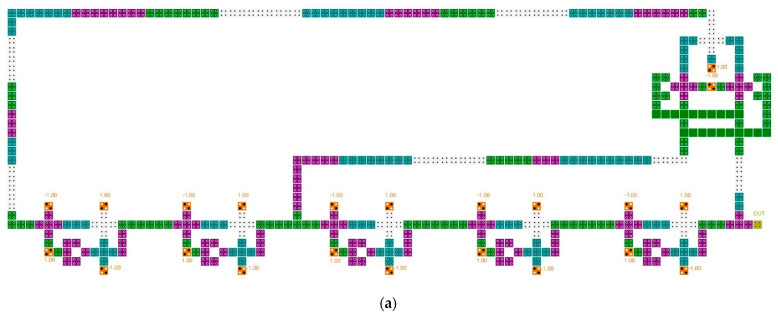

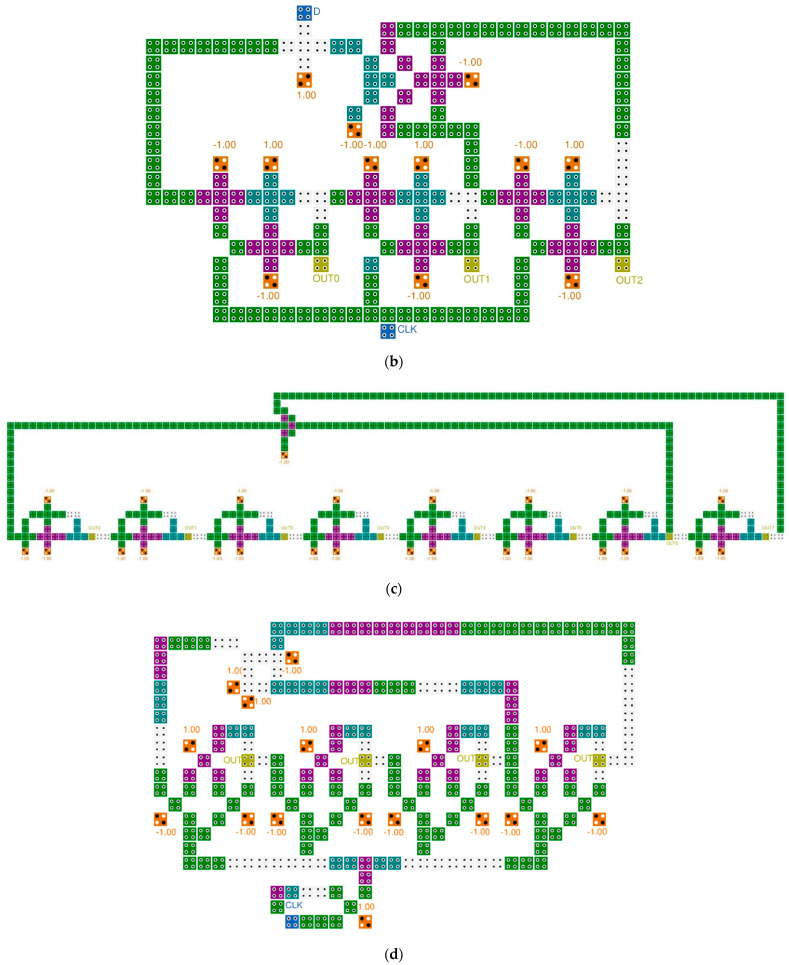
Typical LFSR structures: (**a**) A. Rezaei et al.’s [36]; (**b**) H. Mohammadi et al.’s [37]; (**c**) M. Kaviya et al.’s [38]; and (**d**) Z. Amirzadeh et al.’s [39].

Figure 9 shows typical LFSR structures. The circuit in paper [36] transfers the outputs of the second and fifth blocks in 5-bit SR to the input of the first block after XOR operation. The proposed circuit uses a robust inverter based on the majority vote to increase the signal strength, but the size of the XOR operation is large and there are many wasted areas due to long wiring, which reduces the overall circuit performance. Although the size of each block and XOR has been reduced in the circuit of paper [38], it has similar characteristics to the circuit in paper [36]. The circuit in paper [37] is a 3-bit LFSR structure with a majority vote, and was designed to synchronize the clocks and enable simultaneous output, and strives to minimize the wasted area. Each block of the LFSR structure in paper [39] was designed based on a rotated majority gate, and the required area was minimized by using an XOR gate using cell interaction. In addition, clock synchronization and simultaneous output are possible, and it was designed to enable dual-edge triggering.

## 3. The Proposed Structures

### 3.1. The Proposed 2-to-1 Multiplier and D-Latch

**Figure 10:**
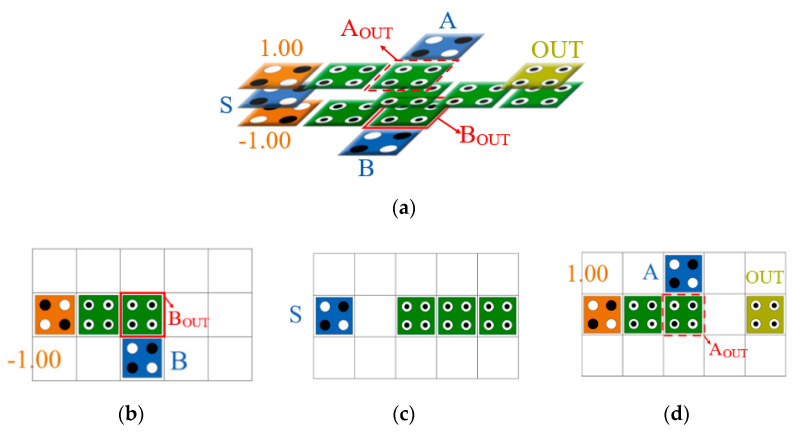
Proposed 2-to-1 Multiplexer: (**a**) full circuit; (**b**) layer 1; (**c**) layer 2; and (**d**) layer 3.

In this paper, we designed a new multilayer 2-to-1 Mux as shown in Figure 10 using cell interaction and a multilayer structure. This structure consists of three layers, and the selection input, S, is placed in the middle layer, and fixed cells of 1 and 0 are placed vertically above and below the selection cell. The input values, A and B, are placed opposite to each other on the third and first layer, respectively, and the output cell, OUT, is placed on the third layer to minimize the required area.

When the S value is −1 (binary logic 0), a signal of +1 is given above and below the S value due to the characteristic of the multilayer structure in which the signal is inverted due to the vertically connected fixed cells, and the output of the fixed cell +1 of the third layer becomes stronger, and the output of the fixed cell −1 on the first floor becomes weaker. Therefore, the output of the A value is stronger than the B value, and the A value is output. Conversely, if the S value is +1 (binary logic 1), the B value is output because the output of the B value is stronger than the A value. In other words, when A_OUT_ and B_OUT_ are determined, this value is set as an output and the function of the Mux is performed normally.

**Figure 11:**
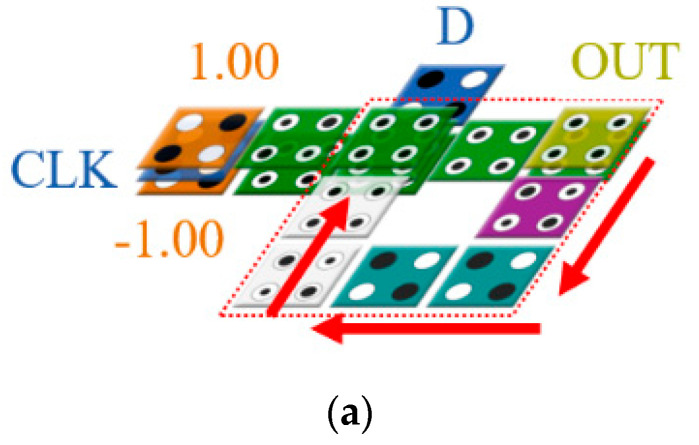

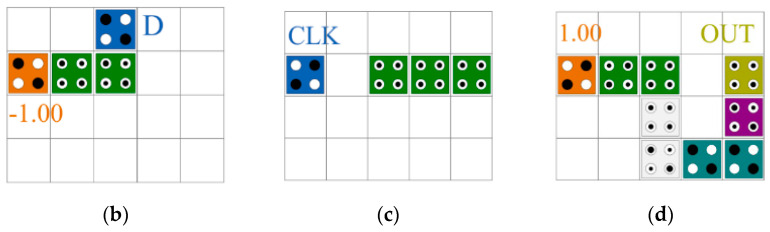
Proposed D-latch: (**a**) full circuit; (**b**) layer 1; (**c**) layer 2; and (**d**) layer 3.

The proposed D-latch is shown in Figure 11. By inputting the output of the proposed 2-to-1 Mux back to the center of the circuit, it is designed to maintain the output value in the circuit. The proposed D-latch was designed to set CLK as the value of the selected cell and maintain the previous output value when the CLK is 0. As shown in Figure 11a, the value from the output cell, OUT, is input to the center cell of the circuit every clock along the arrow. Therefore, depending on the selection of CLK, the input value, D, or the previous output value, OUT(t − 1) is determined as the next output value.

### 3.2. The Proposed 4-Bit SR and LFSR Structure

**Figure 12:**
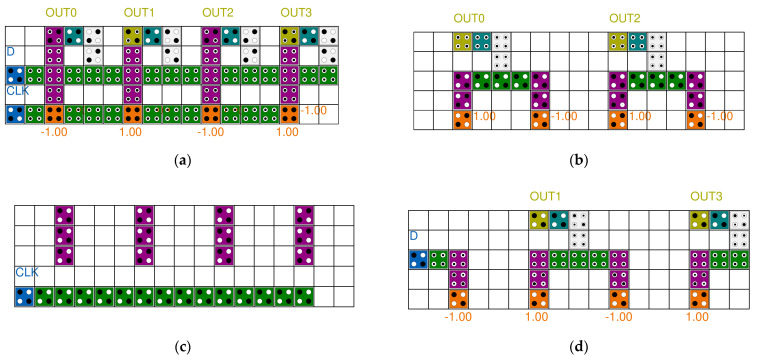
Proposed 4-bit SR: (**a**) Top view; (**b**) layer 1; (**c**) layer 2; and (**d**) layer 3.

Figure 12 shows an SR designed by connecting D-latches. The proposed 4-bit SR has four D-latches, and the input values D and CLK are placed on the leftmost side, and the value of the D-latch can be shifted to the right according to the input value of CLK. Two input values are input to the second and third floors, respectively, so that they can proceed in parallel, and the output is placed on the first and third floors in consideration of the characteristics of the multilayer structure, thereby maintaining the modularity of the circuit.

**Figure 13:**
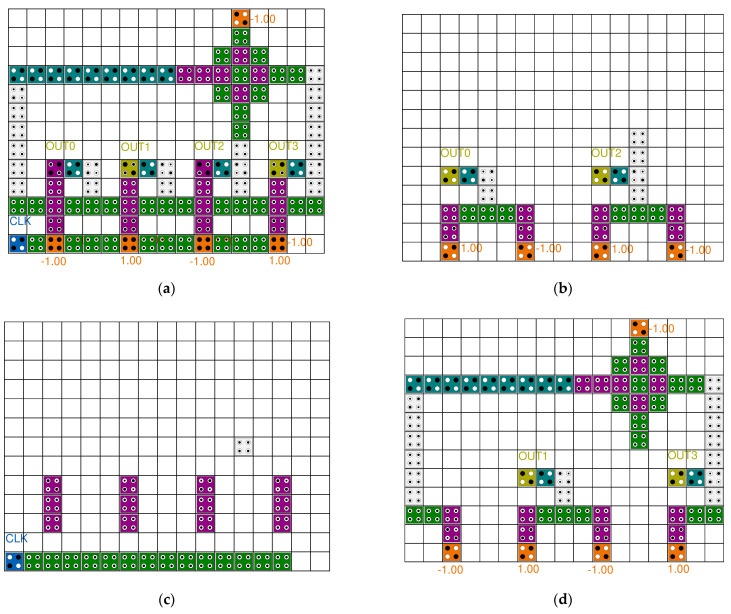
Proposed 4-bit LFSR structure: (**a**) Top view; (**b**) layer 1; (**c**) layer 2; and (**d**) layer 3.

**Figure 14:**
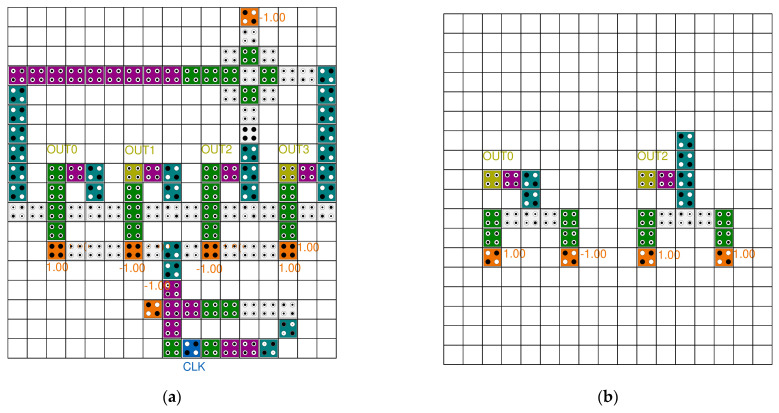

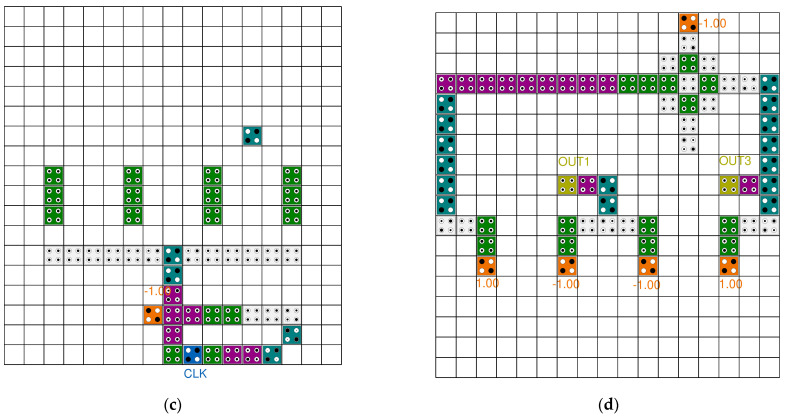
Proposed 4-bit level triggered LFSR structure: (**a**) Top view; (**b**) layer 1; (**c**) layer 2; and (**d**) layer 3.

Figure 13 shows the proposed 4-bit LFSR structure. It was designed based on the logic diagram introduced in Figure 8 using the 4-bit SR proposed in Figure 12 and the XOR gate proposed in Ref. [10]. A level to edge converter was additionally designed in Figure 14 so that the D-latch can operate as a D flip-flop. This allows the circuit to decide which flip-flop can be either negative or positive edge triggered.

Unpredictable random values created through the LFSR structure can be easily converted into ciphertext through plaintext and XOR operation. It can also be returned to plaintext through the same reverse operation. It is used as the simplest and most secure stream cipher.

## 4. Simulation Results and Analyses

In this section, we present the superiority of the proposed structures by analyzing the simulation results of these structures and comparing them with the latest excellent structures.

### 4.1. Structural Analysis

The proposed structures were designed and simulated using QCADesigner version 2.0.3. For the simulation, bistable approximation and a coherence vector simulation engine were used, and the parameters used are shown in Table 4.

**Figure 15:**
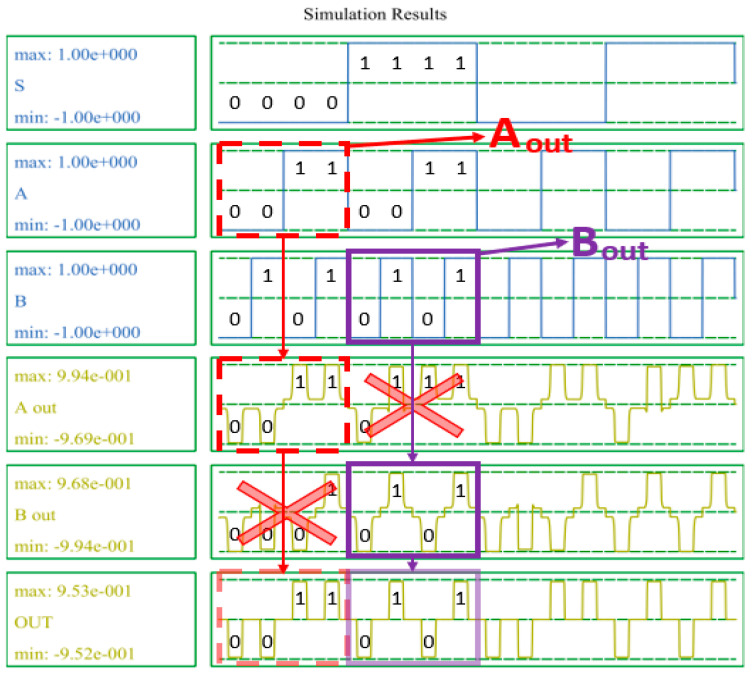
Simulation result of the proposed 2-to-1 Mux.

**Figure 16:**
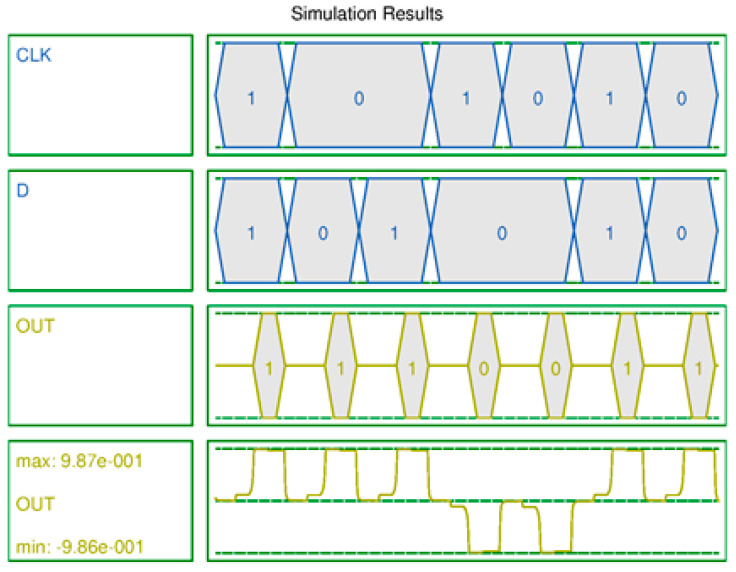
Simulation result of the proposed D-latch.

Figure 15 shows the simulation results of the 2-to-1 Mux. The proposed Mux outputs either an input value A to OUT through A_out_ or an input value B to OUT through B_out_, depending on whether the selection input value, S, is 0 or 1. Figure 16 shows the simulation results of the proposed D-latch. As shown in the truth table in Table 3, when CLK = 1, if the input value D = 1, the output value OUT = 1, and when CLK = 0, the output value maintains the previous output value OUT = 1, regardless of the input value.

**Figure 17:**
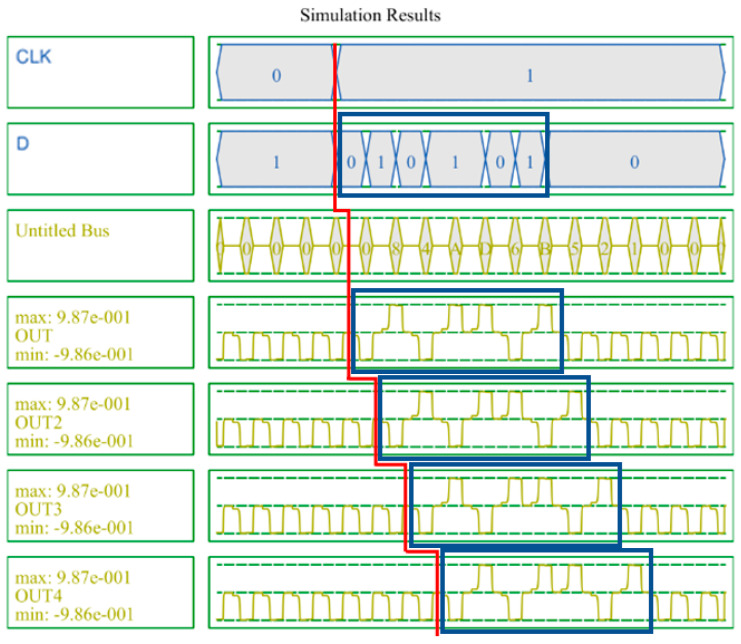
Simulation result of the proposed 4-bit SR.

**Figure 18:**
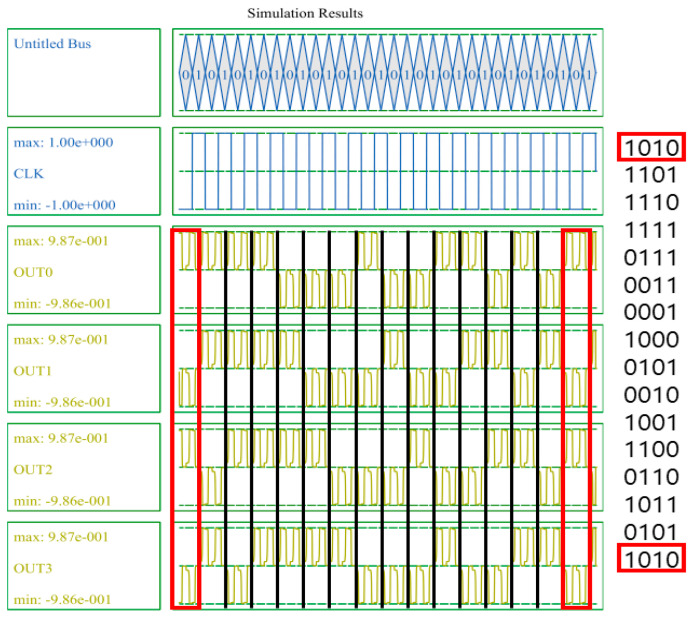
Simulation result of the proposed 4-bit LFSR structure.

Figure 17 shows the simulation results of the SR. A 4-bit SR looks like four D-latches joined together. An input signal D and a clock signal CLK exist, and there are four outputs. These four output signals proceed by passing the previous signal in turn. It can be seen that the values in the blue box are sequentially transferred to the next output value. Figure 18 shows the simulation results of the LFSR structure. When CLK changes from 0 to 1, the value changes, and after 15 clocks have passed, it can be seen that it returns to the initial output value in red box. Therefore, it can be confirmed that the proposed LFSR structure has the maximum period that 4 bits can have.

### 4.2. Performance Comparison

Table 5 compares the performance of the QCA 2-to-1 Mux proposed in this paper with the most recent excellent structures. The criteria for comparison are cell count, circuit area, delay time, and the cost calculated as Area × Latency. In addition, it was described whether the structure was single-layered or multilayered.

Among the latest studies, the single-layered Mux proposed in paper [12] has the highest cost at 12,482, and the single-layered circuit using the cell interaction proposed in paper [17] has the lowest cost of 1421. The proposed multilayered Mux has the same cost as the existing lowest-cost circuit, and shows up to 8.8 times better performance compared to the existing circuit. Additionally, it has excellent circuit scalability due to its symmetrical structure.

As shown in Table 6, the cost of D-latch, which has the best performance, is 6903 in paper [24] among the latest studies. The cost of the proposed structures showed the best performance as 3822, and a performance improvement of about 45% compared to the best typical structure, and up to 4.9 times better performance compared to the existing circuit.

Table 7 and Table 8 compare the performance of the proposed QCA SR and LFSR with the latest and best studies. The cost of the entire circuit can be calculated as Area × Latency, but as the number of bits is different for each circuit, the cost for each bit is calculated again for more objective comparison. In this case, the values after the decimal point are rounded off. It also indicates whether the input and output are in series or parallel.

Among the existing studies, it was confirmed that the multilayered SR of paper [31] had the best performance. It was confirmed that the proposed SR showed superior performance, primarily by 4.5 times, and rising up to 20 times, compared to the recent excellent studies. We have proposed two LFSRs using D-latch and D-F/F, respectively. Compared to the structure using the D-latch, the performance of our D-latch-based structure was 5.3 times to 14.7 times superior, and the D-F/F based structure showed a more than three times improvement in performance compared to the existing best structure.

### 4.3. Energy Dissipation Analysis

The QCADesigner-E [41] tool was used to estimate energy dissipation. QCADesigner-E is an extension of QCADesigner (version 2.0.3) and was developed by the University of Bremen [42]. This application for estimating the power dissipation of QCA circuits is based on the previous studies of Timer and Lent [43,44]. Another tool for estimating power dissipation is QCAPro [45], but it does not calculate the energy dissipation of multilayer structures. Therefore, in this study, QCADesigner-E, which makes it easy to calculate the energy loss of all circuits, was used.

In total energy dissipation and average energy dissipation per cycle, the D-latch-based LFSR structure was 2.2 to 2.4 times superior, and the D-F/F-based LFSR structure showed a performance improvement of more than 20%. As the bit sizes of the existing papers are different, all circuits are increased or decreased at the same rate based on 4 bits for objective comparison. The values in parentheses in Table 9 represent the energy dissipation of the original circuit.

## 5. Conclusions

In this paper, a 2-to-1 Mux and D-latch with wiring were designed. In addition, we proposed a 4-bit SR with D-latch as each block, and a 4-bit LFSR structure by adding an XOR operation. Additionally, by adding an edge trigger, it was possible to extract the output value according to the change of the clock. The study was designed with a multilayer structure and cell interaction, and the performance was analyzed using QCADesigner, and energy dissipation was obtained using QCADesigner-E. The proposed LFSR structure based on D-latch improved performance by about 5.3 times and reduced energy dissipation by 2.2 times compared to the existing best structure. The proposed LFSR structure based on D-F/F showed that performance was improved more than three times and energy dissipation was reduced by more than 20%. This study proposed a QCA LFSR structure that can be used effectively for quantum random number generation (QRNG). QRNG is essential for cryptographic purposes, and can be used directly in stream ciphers and generating quantum random values in cryptographic protocols. In addition to this, QRNG is required in various fields in quantum computing environments.

## Figures and Tables

**Table 1:** Performance comparison of coplanar inverter and multi-layer inverter.

Inverters	Cell Count	Area (nm^2^)	Signal Strength (10^−1^ J)	Structure
Figure 2a	2	1444	5.62	Coplanar
Figure 2b	7	4758	7.75	Coplanar
Figure 3b	2	324	9.69	Multi-layer
Figure 3c	7	1404	8.42	Multi-layer

**Table 2:** Truth table of 2-to-1 Multiplexer.

S	A	B	OUT
0	0	0	0
	0	1	0
	1	0	1
	1	1	1
1	0	0	0
	0	1	1
	1	0	0
	1	1	1

**Table 3:** Truth table of D-latch.

CLK	D	OUT
0	0	OUT(t − 1)
	1	OUT(t − 1)
1	0	0
	1	1

**Table 4:** Simulation parameters.

Parameters	Bistable Approximation	Coherence Vector
Cell size	18 nm	18 nm
Dot diameter	5 nm	5 nm
Cell separation	2 nm	2 nm
Layer separation	11.5 nm	11.5 nm
Clock high	9.8 × 10^−22^ J	9.8 × 10^−22^ J
Clock low	3.8 × 10^−23^ J	3.8 × 10^−23^ J
Clock shift	0	0
Clock amplitude factor	2.0	2.0
Relative permittivity	12.9	12.9
Radius of effect	65 nm	80 nm

**Table 5:** Performance comparison of QCA 2-to-1 Mux.

Circuit	Cell Count	Area (nm^2^)	Latency (Clock Cycle)	Cost (Area × Latency)	Structure
[12]	23	24,964	0.50	12,482	coplanar
[13]	12	9604	0.25	2401	coplanar
[14]	15	16,284	0.50	8142	coplanar
[15]	21	9604	0.75	7203	multi-layer
[16]	9	7644	0.25	1911	coplanar
[17]	9	5684	0.25	1421	coplanar
[18]	10	4524	0.50	2262	coplanar
Figure 10	13	5684	0.25	1421	multi-layer

**Table 6:** Performance comparison of QCA D-latch.

Circuit	Cell Count	Area (nm^2^)	Latency (Clock Cycle)	Cost (Area × Latency)	Structure
[20]	28	24,964	0.50	12,482	coplanar
[21]	19	16,284	0.75	12,213	coplanar
[22]	23	21,804	0.75	16,353	coplanar
[23]	24	18,644	1.00	18,644	coplanar
[24]	13	9204	0.75	6903	coplanar
[25]	18	9204	1.00	9204	coplanar
[26]	27	13,924	1.00	13,924	multilayer
Figure 11	17	7644	0.50	3822	multilayer

**Table 7:** Performance comparison of QCA SR.

Circuit	Cell Count	Area (nm^2^)	Latency (Clock Cycle)	Cost (Area × Latency)	Bits	Cost/bit	Type	Structure
[28]	102	81,844	3.00	245,532	3	81,844	SISO	coplanar
[29]	127	108,564	3.00	325,692	3	108,564	SISO	coplanar
[30]	105	134,524	2.75	369,944	3	123,315	SISO	coplanar
[31]	120	28,124	3.00	84,372	3	28,124	SISO	multilayer
[32]	92	68,724	3.75	257,715	4	64,429	SIPO	coplanar
[33]	177	149,124	2.00	298,248	3	99,416	PIPO	coplanar
Figure 12	80	33,124	0.75	24,843	4	6210	SIPO	multilayer

**Table 8:** Performance comparison of QCA LFSR structure.

Circuit	Cell Count	Area (nm^2^)	Latency (Clock Cycle)	Cost (Area ✕ Latency)	Bits	Cost/bit	Type	Structure
[36]	440	958,324	1.25	1,197,905	5	239,581	Latch	multilayer
[37]	191	230,044	1.25	287,555	3	95,852	Latch	coplanar
[38]	472	918,924	0.75	689,193	8	86,149	Latch	coplanar
[39]	226	275,044	2.00	550,088	4	137,522	F/F	coplanar
Figure 13	120	87,204	0.75	65,403	4	16,351	Latch	multilayer
Figure 14	136	121,004	1.50	181,506	4	45,377	F/F	multilayer

**Table 9:** Energy dissipation comparison of QCA 4-bit LFSR structure.

Energy Dissipation	[36]	[37]	[38]	[39]	Figure 13	Figure 14
Total (*e*^−2^ eV)	7.61 (9.51)	7.92 (5.94)	7.20 (14.4)	4.35	3.33	3.62
Average per cycle (*e*^−3^ eV)	6.91 (8.64)	7.20 (5.40)	6.55 (13.1)	3.95	3.03	3.29

## Data Availability

The data presented in this study are available on request from the authors.
